# In situ observation of the atomic shuffles during the {${{11}}\bar{{{2}}}{{1}}$} twinning in hexagonal close-packed rhenium

**DOI:** 10.1038/s41467-024-47343-z

**Published:** 2024-04-06

**Authors:** Yang He, Zhengwu Fang, Chongmin Wang, Guofeng Wang, Scott X. Mao

**Affiliations:** 1 Department of Mechanical Engineering and Materials Science, University of Pittsburgh, Pittsburgh, PA 15261 USA; 2 Environmental Molecular Sciences Laboratory, Pacific Northwest National Laboratory, Richland, WA 99352 USA

**Keywords:** Nanoscale materials, Structural materials

## Abstract

Twinning, on par with dislocations, is critically required in plastic deformation of hexagonal close-packed crystals at low temperatures. In contrast to that in cubic-structured crystals, twinning in hexagonal close-packed crystals requires atomic shuffles in addition to shear. Though the twinning shear that is carried by twinning dislocations has been captured for decades, direct experimental observation of the atomic shuffles, especially when the shuffling mode is not unique and does not confine to the plane of shear, remains a formidable challenge to date. Here, by using in-situ transmission electron microscopy, we directly capture the atomic mechanism of the $\left\{11\bar{2}1\right\}$ twinning in hexagonal close packed rhenium nanocrystals. Results show that the $\left\{11\bar{2}1\right\}$ twinning is dominated by the (**b**_1/2_, h_1/2_) twinning disconnections. In contrast to conventional expectations, the atomic shuffles accompanying the twinning disconnections proceed on alternative basal planes along 1/6 $\left\langle 1\bar{1}00\right\rangle$, which may be attributed to the free surface in nanocrystal samples, leading to a lack of mirror symmetry across the $\left\{11\bar{2}1\right\}$ twin boundary.

## Abstract (web summary)

Atomic shuffles are important phenomena accompanying the twinning in hexagonal close-packed crystals, but the direct experimental observation on it is lacking. Here, the authors show the shuffling mechanism of {112 ®1} twinning in rhenium nanocrystals by in situ transmission electron microscopy.

## Introduction

Due to a lack of easy slip systems, twinning is extensively activated and plays a vital role in low-temperature deformation of hexagonal close-packed (HCP) metals, typically such as magnesium^1^. Therefore, the alloy design and processing of HCP metals inevitably need to consider twinning, drawing intense interest in the fundamental mechanisms of twinning in HCP crystals.

In contrast to the face-centered cubic structures, which contain only one atom at each Bravais lattice point, the HCP structure has two atoms at each lattice point; as such, in addition to the homogeneous shear, angstrom-scale shuffles of atoms are also needed to move both atoms in the motif of the parent crystal to their corresponding positions in the twin. As such, twinning disconnections with both steps and dislocations features are generally accepted to describe the interfacial defects on twin boundaries in HCP crystals. This intrinsic complexity poses significant challenges to the experimental resolution of the twinning mechanism, especially when the shuffling mode is not unique and is not confined to the plane of shear. Theoretical works in this regard are largely based on topological analysis and atomistic simulations^2–4^; the results of which can vary significantly depending on the interatomic potentials and boundary conditions^5,6^, leading to many controversies, for instances, on the $\left\{10\bar{1}2\right\}$ twinning and the $\left\{11\bar{2}2\right\}$ twinning mechanisms^7–11^. Previous experimental investigations on the twinning mechanism, as have been done in face-centered cubic metals^12^, largely focus on imaging the twinning dislocations^13–16^ on the plane of shear, which normally is not sufficient to discern the atomic shuffles.

A typical example is the $\left\{11\bar{2}1\right\}$ twinning which, on par with the $\left\{10\bar{1}2\right\}$ twinning, is a common tension twinning mode in HCP crystals and plays a significant role in the mechanical behaviors of Ti^17^, Zr^18^, Co^19^, and Re^20^. Different from the most prevalent $\left\{10\bar{1}2\right\}$ twinning wherein the required atomic shuffles proceed on the plane of shear^7,16,21,22^, the atomic shuffles accompanying the $\left\{11\bar{2}1\right\}$ twinning likely proceed perpendicular to the plane of shear^2,4^; it has been reported by A. Serra et al. in several works that molecular dynamics simulations indicate that the most likely directions of shuffle in $\left\{11\bar{2}1\right\}$ twinning are actually not in the plane of shear but on the twinning plane and perpendicular to the twinning shear direction. Therefore, characterizing the twinning dislocations cannot pinpoint the exact shuffling mode^14,15^.

**Fig. 1:**
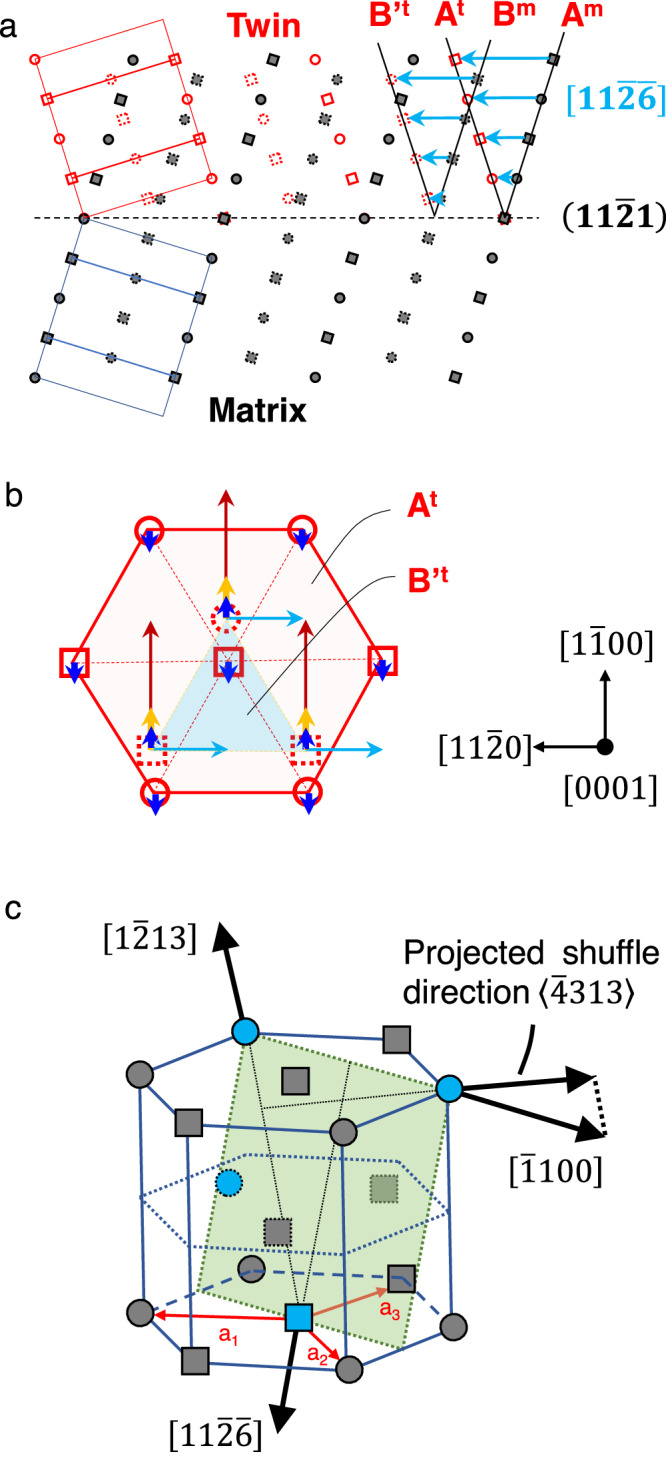
Topological analysis of the potential twinning shuffles in $\left\{11\bar{2}1\right\}$ twinning of the HCP metals. **a** Dichromatic pattern for the $\left(11\bar{2}1\right)$ twinning viewed along the $\left[1\bar{1}00\right]$ direction. The filled gray circles and squares represent atoms in the matrix; the open red circles and squares represent atoms in the twin. Solid circles, dash circles, solid squares, and dash squares indicate atoms of different levels along the $\left[1\bar{1}00\right]$ direction. Stacking sequence of basal planes in the matrix A^m^B^m^A^m^B^m^ changes to A^t^B’^t^A^t^B’^t^ after the homogeneous shear (cyan arrows). **b** A^t^B’^t^A^t^B’^t^ stacking sequence of the basal planes viewed along the $\left[0001\right]$ direction of the twin. Arrows with the same color indicate one shuffling mode; there are a total of four shuffling modes. **c** The $\left(11\bar{2}1\right)$ plane (green-colored) in a HCP unit cell. Atoms belong to two $\left(22\bar{4}2\right)$ sub-planes and are colored blue with solid outlines and dash outlines, respectively.

As shown in the dichromatic complex in Fig. 1a and b, the homogeneous shear during the $\left\{11\bar{2}1\right\}$ twinning changes the ABAB stacking sequence of the basal planes; and restoring the correct stacking sequence of the basal planes requires atomic shuffles according to one of the four shuffling modes^4,23^. As illustrated in Fig. 1b, the light blue arrows indicate that alternative basal planes shuffle along 1/6 $\left[11\bar{2}0\right]$ which can be captured by imaging the process on the plane of shear. The other three shuffling modes require atoms to move along the $\left[1\bar{1}00\right]$ direction, i.e., perpendicular to the plane of shear. Specifically, the yellow arrows indicate alternative basal planes shuffle along 1/6 $\left[1\bar{1}00\right]$. The blue arrows denote neighboring basal planes shuffle in opposite directions along 1/12 $\left[1\bar{1}00\right]$ and 1/12 $\left[\bar{1}100\right]$, respectively. The red arrows denote alternative basal planes shuffle along 1/2 $\left[1\bar{1}00\right]$. Unfortunately, due to the difficulty in detecting the angstrom-scale process in the complex HCP structure, the key features of the dominant twinning dislocation along with the atomic shuffles in $\left\{11\bar{2}1\right\}$ twinning remain theoretical conjectures to date.

Here, by using advanced in-situ aberration-corrected transmission electron microscopy (TEM) and delicate crystal manipulation techniques^14,24^, the active twinning disconnections and shuffling mode in the $\left\{11\bar{2}1\right\}$ twinning process are explicitly unveiled. We show that $\left\{11\bar{2}1\right\}$ twinning in HCP rhenium nanocrystals is dominated by the (**b**_**1/2**_, h_1/2_) twinning disconnections, and corresponding atomic shuffles proceed on every other basal plane along 1/6 $\left\langle 1\bar{1}00\right\rangle$, resulting in a lack of mirror symmetry across the $\left\{11\bar{2}1\right\}$ twin boundary. This work provides a viable route to study the complex and angstrom-scale atomic shuffles in HCP crystals.

## Results

### In-situ observation of $\left\{{{{{{\bf{11}}}}}}\bar{{{{{{\bf{2}}}}}}}{{{{{\bf{1}}}}}}\right\}$ twinning on the plane of shear

**Fig. 2:**
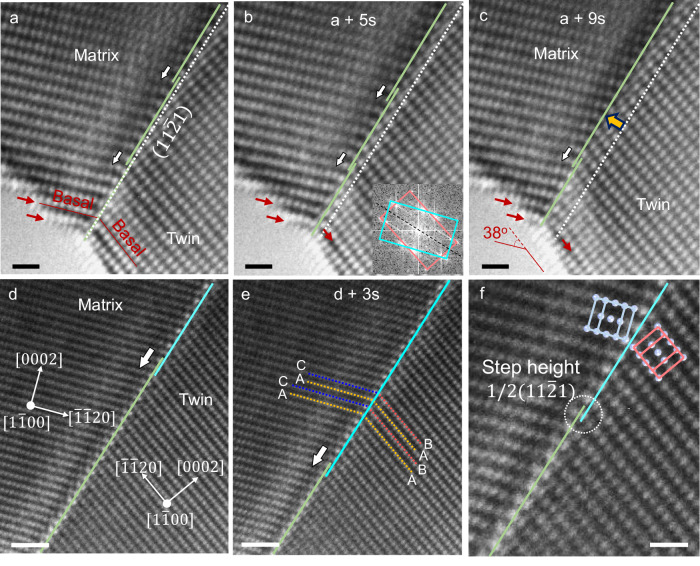
In-situ atomic resolution TEM observation of the $\{11\bar{2}1\}$ twinning and detwinning processes on the plane of shear. The Re crystal was under $\langle11\overline{2}13\rangle$-oriented tension and compression during the twinning and detwinning, respectively. **a**–**c** Sequential TEM snapshots showing the twinning process. Orange-colored solid lines indicate the $\left(11\bar{2}1\right)$ twin boundary. White lines indicate the original position of the twin boundary. Red arrows indicate the surface facets' evolution during the twinning process. Inset in (**b**) shows the FFT of the twinning region, showing an orientation relation that is consistent with that of a $\left\{11\bar{2}1\right\}$ twin; therein, the pink and cyan squares indicate the reciprocal lattices of the twin and matrix, respectively. **d** and **e** Sequential TEM snapshots showing the detwinning process. Sections of the $\left(11\bar{2}1\right)$ twin boundaries are indicated by lines with different colors. White arrows in **a**–**e** indicate the migration direction of steps (twinning disconnections) on the twin boundary. **f** Enlarged atomic resolution image of a twinning dislocation. The white circle indicates the twinning disconnection. Inset shows the unit cells of the matrix and the twin. Scale bar: **a**–**c** 500 pm, **d**, **e** 1 nm, **f** 500 pm.

A Re single crystal is initially oriented along the $\left\langle 1\bar{1}00\right\rangle$ direction. Tension is applied along the $\left\langle 11\bar{2}13\right\rangle$ direction such that the $\left\{11\bar{2}1\right\}$ twinning shear has a much larger Schmid factor than the competing deformation mode of basal slip. Consequently, plastic deformation of the Re crystal is primarily mediated by the $\left\{11\bar{2}1\right\}$ twinning (Fig. 2a–c). During twin growth, the basal surface facets of the parent crystal are transformed into basal surface facets of the twin (see red arrows in Fig. 2a–c), indicating that the basal plane is the conjugate twinning plane. Based on the attendant surface inclination (Fig. 2c), the twinning shear is calculated to be ~0.65 along the $\left\langle \bar{1}\bar{1}26\right\rangle$ direction. Above all, the twinning elements here are K_1_ $\left\{11\bar{2}1\right\}$, K_2_ $\left(0001\right)$, η_1_ $\left\langle \bar{1}\bar{1}26\right\rangle$, η_2_ $\left\langle 11\bar{2}0\right\rangle$, which are consistent with those of the classical $\left\{11\bar{2}1\right\}$ twinning. The interfacial defects that have mediated the twin boundary migration in both twinning (Fig. 2a-c) and detwinning (Fig. 2d, e and Video 1) show a universal step height of 1/2$\left\{11\bar{2}1\right\}$ plane, indicating that they are (b_1/2_, h_1/2_) twinning disconnections (see Fig. 2f). Moreover, no evident atomic shuffle is detected in this observation. Therefore, the shuffling mode within the plane of shear can be excluded, and the atomic shuffles associated with the $\left\{11\bar{2}1\right\}$ twinning here should be along the $\left\langle 1\bar{1}00\right\rangle$ direction^4^.

### In-situ observation of $\left\{{{{{{\bf{11}}}}}}\bar{{{{{{\bf{2}}}}}}}{{{{{\bf{1}}}}}}\right\}$ twinning along the $\left\langle {{{{{\bf{11}}}}}}\bar{{{{{{\bf{2}}}}}}}\bar{{{{{{\bf{3}}}}}}}\right\rangle$ direction

**Fig. 3:**
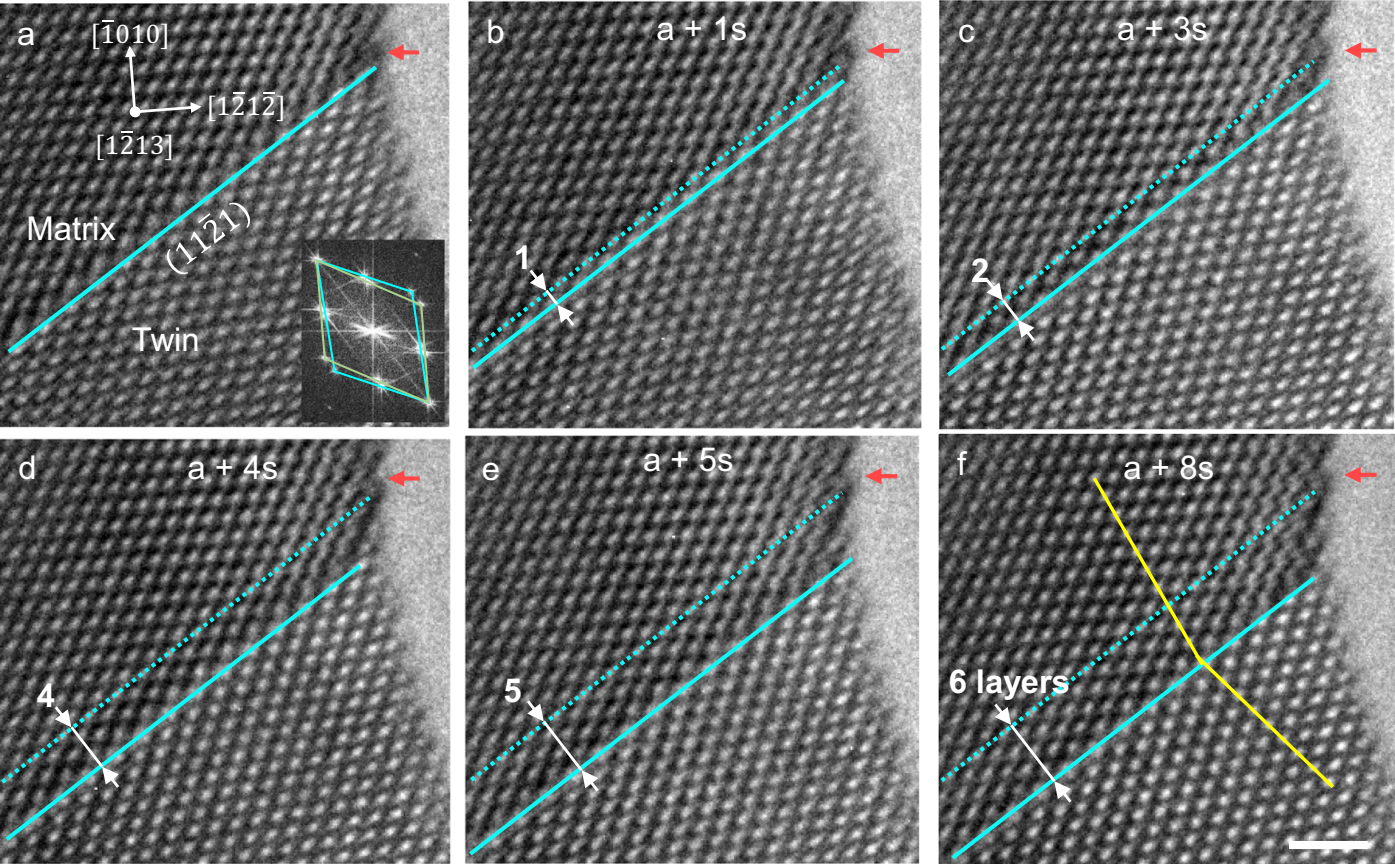
In-situ high-resolution TEM observation of the $\left\{11\bar{2}1\right\}$ detwinning processes in the $\left\langle1\bar{2}13\right\rangle$ direction. **a**–**f** Sequential TEM snapshots of the process. Red arrows indicate the reference point. Solid cyan lines indicate the $\left(11\bar{2}1\right)$ twin boundary. Dash cyan lines in **b**–**f** indicate the original position of the twin boundary in panel (**a**). Inset in (**a**) shows the FFT of the twinning region, showing an orientation relation that is consistent with that of a $\left\{11\bar{2}1\right\}$ twin; therein, the pink and cyan squares indicate the reciprocal lattices of the twin and matrix, respectively. The yellow lines in panel (**f**) mark the edges of the prismatic planes in the matrix and twin. Scale bar: 1 nm.

Figure 3 and Video 2 show the high-resolution $\left\langle 1\bar{2}13\right\rangle$-view of the $\left\{11\bar{2}1\right\}$ twinning process in another Re nanocrystal. Compressive loading is directed along the $\left[\bar{6}51\bar{1}\right]$ direction of the crystal to activate the $\left(11\bar{2}1\right)$ twinning. As shown in Fig. 3a–f, the twin boundary propagates in a layer-by-layer manner, implying that the dominating twinning disconnection should have a step height of 1 or 1/2 $\left\{11\bar{2}1\right\}$ layer. It should be noted that the (**b**_**1**_, h_1_), if present, would dissociate into two (**b**_**1/2**_, h_1/2_) disconnections since the dissociation can effectively reduce the long-range elastic strain energy^2,25^. Additionally, since no twinning disconnection with a step height of one $\left\{11\bar{2}1\right\}$ layer is directly captured throughout the observation, the dominant twinning disconnections are more likely the (**b**_**1/2**_, h_1/2_). This finding is consistent with the prior observations (see Fig. 2f).

### Pin-point the shuffling mode

**Fig. 4:**
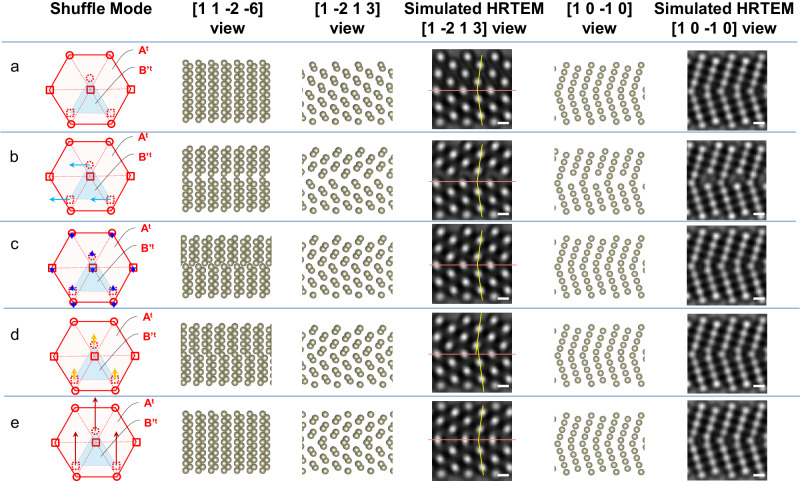
Atomic models constructed based on different shuffling mechanisms and corresponding simulated HRTEM images showing projection views along the $\left[1\bar{2}13\right]$ and $\left[\bar{1}100\right]$ directions. **a** No shuffle is applied after the homogeneous shear. **b** Shuffle proceeds on every other basal plane along 1/6 $\left\langle 11\bar{2}0\right\rangle$ (light blue arrows). **c** The conventional shuffling mechanism wherein adjacent basal planes shuffle in opposite directions along 1/12 $\left\langle 1\bar{1}00\right\rangle$ (blue arrows). **d** The observed shuffling mechanism wherein every other basal plane shuffle 1/6 $\left\langle 1\bar{1}00\right\rangle$ (yellow arrows). **e** Shuffling mechanism in which every other basal plane shuffle 1/2 $\left\langle 1\bar{1}00\right\rangle$ (red arrows). The red lines in the simulated HRTEM images indicate the $\{11\bar{2}1\}$ twin boundaries. The yellow lines mark the edges of the prismatic planes in the matrix and twin. Scale bar: 200 pm.

To further identify the atomic shuffles along the $\left\langle \bar{1}100\right\rangle$ direction, quantitative analysis was carried out on the TEM snapshot of the $\left\{11\bar{2}1\right\}$ twin boundary during its propagation. As shown in Fig. 4, atomic structures of the $\left\{11\bar{2}1\right\}$ twin are constructed based on different shuffling mechanisms as shown in Fig. 1: Model **a** involves no shuffle, wherein the atomic structure after the homogeneous shear is not an HCP structure (i.e., not in ABAB… stacking sequence); Model **b** represents the shuffle of alternative basal planes along $1/6\left[11\bar{2}0\right]$, which moves corresponding atoms away from the twin boundary and causes the hollow areas as reflected by the observation from the $\left[\bar{1}100\right]$ view; Models **c** and **d** have the same net magnitude of shuffle, i.e., $\left|1/6\left\langle 1\bar{1}00\right\rangle \right|$, which both change the ABAB… stacking sequence in the matrix into the ACAC… sequence in the twin, and cause the lack of mirror symmetry on the $\left\{11\bar{2}1\right\}$ twin boundary from the $\left[11\bar{2}\bar{6}\right]$ view; Model **e** involves large shuffle of alternative basal planes along 1/2$\left\langle 1\bar{1}00\right\rangle$ without breaking the mirror symmetry on $\left\{11\bar{2}1\right\}$ twin boundary. Then, these atomic structures are used as input for simulating high resolution TEM (HRTEM) images of the twin viewed along the $\left[1\bar{2}13\right]$ direction and the $\left[\bar{1}100\right]$ direction. Note that we employ a multi-slicing method for the TEM image simulation, and all parameters used for the simulation are reasonable estimations of the experiment. Specifically, the TEM accelerating voltage is 300 kV, spherical aberration is 20 nm, defocus is 25 nm, and the thickness of the sample is 12 nm. The simulated HRTEM images are then compared with the enlarged experimental HRTEM snapshots in Figs. 2, 3.

**Fig. 5:**
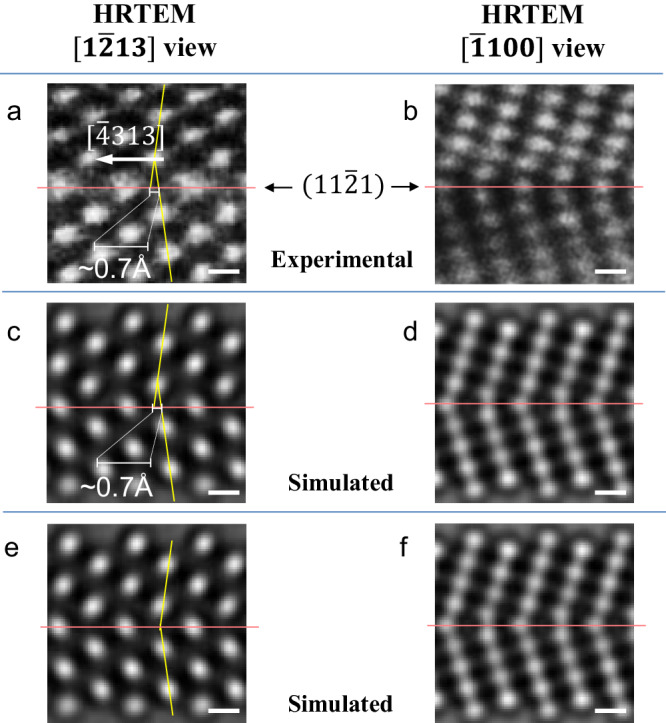
Quantitative comparison between the in-situ experimental HRTEM images (a, b) and the simulated HRTEM images (c–f) based on structure models derived from the observed shuffling mechanism (c, d) and the shuffling mechanism commonly referred to in literature^23,34^ (e, f), to determine the shuffling mechanism. The red lines in the simulated HRTEM images indicate the $\left\{11\bar{2}1\right\}$ twin boundaries. The yellow lines mark the edges of the prismatic planes in the matrix and twin. These lines are just for illustration purpose, and their position are determined by using the intensity peaks of atom columns within the referred planes. Scale bar: 200 pm.

First of all, the simulated HRTEM image of the projection view along the $\left[\bar{1}100\right]$ direction in Fig. 4b is obviously different from the experimental image, as shown in Fig. 2, evidencing that the shuffling mechanism wherein every other basal plane shuffle $1/6\left[11\bar{2}0\right]$ is not active. Note that based on the simulated HRTEM images for $\left[\bar{1}100\right]$ projection view, shuffling mechanisms along the $\left[\bar{1}100\right]$ direction cannot be determined. Referring to the projection view along the $\left[1\bar{2}13\right]$ direction, if the atomic shuffles proceed as those shown in Fig. 4a–c and e, the edges of the prismatic planes in the matrix and the twin (as marked by the yellow lines in these panels) should intersect exactly at the twin boundary. By contrast, the in-situ HRTEM observations in Fig. 3 clearly show otherwise (see the enlarged image in Fig. 5a); the prismatic planes in the matrix and the twin do not intersect exactly at the $\left\{11\bar{2}1\right\}$ twin boundary. As shown in Fig. 5, by comparing the experimental snapshots (Fig. 5a and b) with the simulated HRTEM images along the $\left[1\bar{2}13\right]$ direction (Fig. 5c or  4d) and the $\left[\bar{1}100\right]$ direction (Fig. 5d or  4d), the quantitative match between the experimental and simulated HRTEM images is found, evidencing that the alternate basal planes shuffle 0.8 Å along the $\left[1\bar{1}00\right]$ direction, i.e., 1/6 $\left[1\bar{1}00\right]$. Viewing along the $\left\langle 1\bar{2}1\bar{3}\right\rangle$ direction, this atomic shuffle should have a projected value of ~0.7 Å along the $\left\langle \bar{4}313\right\rangle$ direction, which is quantitatively consistent with the measurement from the in-situ TEM snapshot. On the other hand, as evidenced by the obvious difference between the experimental HRTEM image in Fig. 5a and the simulated HRTEM image in Fig. 5e, the conventional shuffling mechanism, in which adjacent basal planes shuffle in opposite directions along 1/12 $\left[1\bar{1}00\right]$ and 1/12 $\left[\bar{1}100\right]$, is apparently not what has been observed in our in-situ experiments.

## Discussion

The above observations directly captured the atomic shuffles in the $\left\{11\bar{2}1\right\}$ twinning. Note that classical twinning theory indicates that twinning disconnection on the $\left\{11\bar{2}1\right\}$ plane should have a step height of one $\left\{11\bar{2}1\right\}$ layer^1,26^. Molecular dynamics simulations show that such dislocation tends to decompose into two dislocations with an identical step height of 1/2 $\left\{11\bar{2}1\right\}$ layer and expanded cores^2,4,25^. Our observation here provides direct evidence of the simulation results.

The observed shuffling mode is different from the simulation results wherein atomic shuffles have minimum magnitude (i.e. neighboring basal planes shuffle in the opposite directions, along ∓1/12$\left\langle 1\bar{1}00\right\rangle$)^2,27^. This difference may be attributed to the different boundary conditions in simulations and in experiments. When the twinning disconnection is nucleated from the side surface, the surface tension may restrain the prismatic surface facet from bending, and thereafter, the neighboring prismatic plane right beneath the surface facet has to shuffle 1/6 $\left\langle 1\bar{1}00\right\rangle$ (as indicated by the yellow arrows in Fig. 1b). On the other hand, the shuffling scheme in the previous simulations^27^, though entails smaller shuffle magnitude, introduces a bending distortion of the crystal surface. The competition between the relatively large shuffle magnitude and the surface distortion in the actual twinning process apparently has favored the former in our experimental observations. For the shuffling mode in simulation to work in the confined environment in the interior of a bulk sample, strain accommodation mechanisms such as the deformation of the adjacent grains^20^ are required. In other words, if such strain accommodation mechanism on-site is harder to activate than the observed shuffling mode, it is reasonable to expect the observed shuffling mode that does not generate additional strain on the prismatic plane in the interior of the bulk samples. Additionally, though our finding is based on Re, it may be reasonably extended to other bulk HCP metals such as Ti^17^ and Co^19^. Re has been demonstrated to be a suitable model material for the study of twinning in HCP metals^14,20^, wherein the $\left\{11\bar{2}1\right\}$ twinning plays a significant role in deformation. The *c/a* ratio of Re (1.615) is very close to that of Ti (1.588); and hence it is reasonable to expect a similar deformation mode in them^28^. Nevertheless, further works are needed to validate the atomic shuffle mechanism in other and bulk HCP metals. As has been demonstrated in this work, this could be achieved by in situ atomic-scale observations (with possibly thin foil samples) of the twinning process on the two deliberately chosen viewing directions, i.e., $\left\langle 1\bar{2}1\bar{3}\right\rangle$ and $\left\langle \bar{1}100\right\rangle$. Also, it should be noted that the peculiar $\left\{11\bar{2}1\right\}$ twinning in Zr, involving an abnormal twinning shear^18^ and associated mechanisms^3,29^, are far beyond the findings here.

In retrospect, this study demonstrates a viable route to study atomic shuffles in the twinning of HCP crystals by integrating in-situ TEM and delicate crystal manipulation techniques. Atomic shuffles during the $\left\{11\bar{2}1\right\}$ twinning process of HCP crystals are unambiguously revealed. The results demonstrate that, though the twinning elements and the dominant twinning disconnections are consistent with classical expectations, the atomic shuffles in rhenium nanocrystals are totally different from previous expectations^2,25^. An interesting inference of the observed shuffling mode is that the atomic shuffles that are carried by the (**b**_**1/2**_, h_1/2_) twinning disconnections may vary; when one twinning disconnection carries the shuffle of 1/6 $\left\langle \bar{1}100\right\rangle$, the following twinning disconnection will not entail any atomic shuffles. To confirm this point and its prevalence in other materials and bulk counterparts, further simulations and/or experiments on the exact structure of the twin boundary are needed. Moreover, the shuffling mode leads to a lack of mirror symmetry on the $\left\{11\bar{2}1\right\}$ twin boundary (as illustrated in Fig. 4d), which may have an impact on the segregation of alloying elements on the twin boundary^30^; thereby, alloying may interfere with the kinetics of the observed twinning mechanism.

## Methods

### Materials and sample processing

Re nanocrystals are used as model materials throughout this study^20,31–33^. The advantages of using nanocrystals are that high stresses and dislocation-free samples can be attained to trigger the desired deformation mode without interference from the other deformation carriers^34^. In addition, Re is a crucial structural material for high-temperature applications^35^, and has a typical HCP structure that is stable at room temperature in air and highly resistant to 300 keV electron beam irradiation^36,37^. Commercial Re metal (99.98% purity, 0.25 mm-thick plate) is acquired from Sigma-Aldrich (MO, USA). The Re plate was cut into small rods, which were mechanically polished to remove surface oxides, rinsed in ethanol alcohol, and plasma-cleaned to remove contamination on the surface. Then, the Re rod was cut to reveal a fresh surface. Two rods were loaded onto the fixed-end and piezo-end of a Nanofactory-STM holder. The nanotips on the fresh surface were adjusted to the preferred zone axis, manipulated to contact each other, and welded to form bi-crystals. Note that to reveal possible atomic shuffles within and perpendicular to the plane of shear, high-resolution observations on the $\left\{11\bar{2}1\right\}$ twinning process are carried out along the $\left[\bar{1}100\right]$ direction and the $\left[1\bar{2}1\bar{3}\right]$ direction, respectively. Note that both $\left[1\bar{2}1\bar{3}\right]$ and $\left[11\bar{2}\bar{6}\right]$ directions have non-trivial projections along the $\left[\bar{1}100\right]$ direction (see Fig. 1c); the $\left[1\bar{2}1\bar{3}\right]$ view is better than the $\left[11\bar{2}\bar{6}\right]$ view since it offers 2-dimensional lattice images while the $\left[11\bar{2}\bar{6}\right]$ view only offers 1-dimensional fringe images.

### In-situ TEM experimental procedures

Tensile and compression experiments were accomplished by using the piezo-electrical system on the Nanofactory-STM holder at controlled strain rates of ~10^−3^/s. FEI 80–300 Titan ETEM was used for the in-situ TEM experiments. The TEM was equipped with an imaging lens spherical-aberration corrector and had a ~0.10 nm point-to-point resolution at 300 keV after aberration-correction, which was much smaller than the minimum distances between atoms columns in the shear plane projection (~1.38 Å). Moreover, the aberration-correction minimized the “delocalization” effect in the TEM observation, making the TEM perfect for imaging the twin boundaries. During the in-situ straining, the deformation was recorded by using a charge-coupled device at 2 frames per second.

## Data Availability

All data needed to evaluate the findings in the paper are present in the paper. Additional data that support the findings of this study are available from the corresponding author upon request.
